# Synthesis and Electrochemical Evaluation of MSNs-PbAE Nanocontainers for the Controlled Release of Caffeine as a Corrosion Inhibitor

**DOI:** 10.3390/pharmaceutics14122670

**Published:** 2022-11-30

**Authors:** Martín Aguirre-Pulido, Jorge A. González-Sánchez, Luis R. Dzib-Pérez, Montserrat Soria-Castro, Alejandro Ávila-Ortega, William A. Talavera-Pech

**Affiliations:** 1 Corrosion Research Center, Autonomous University of Campeche, Avenida Héroe de Nacozari 480, Campeche 24079, Mexico; 2 Department of Applied Physics, CINVESTAV-IPN Merida Unit, Km 6 Old Road to Progreso, Mérida 97310, Mexico; 3 Faculty of Chemical Engineering, Autonomous University of Yucatán (UADY), Periférico Norte Km 33.5, 13615, Chuburna de Hidalgo Inn, Mérida 97203, Mexico

**Keywords:** MSNs-PbAE, nanocontainers, controlled release, corrosion inhibitor, electrochemical analysis

## Abstract

In this paper, a controlled-release system of caffeine as a corrosion inhibitor was obtained by encapsulating it in MCM-41 silica nanoparticles coated with a poly(β-amino ester) (PbAE), a pH-sensible polymer. Encapsulation was verified using Fourier transform infrared spectroscopy (FTIR) and thermogravimetry (TGA). The release of caffeine from the nanocontainers was analyzed in electrolytes with pH values of 4, 5, and 7 using UV–Vis, showing a 21% higher release in acidic electrolytes than in neutral electrolytes, corroborating its pH sensitivity. Electrochemical impedance spectroscopy (EIS) and potentiodynamic polarization were used to determine the inhibition mode and efficiency of the encapsulated and free caffeine. The caffeine released from the nanocontainers showed the highest efficiency, which was 85.19%. These results indicate that these nanocontainers could have potential use in smart anticorrosion coating applications.

## 1. Introduction

The corrosion process is the irreversible and spontaneous reaction of metals with species in the environment to obtain a more stable thermodynamic condition in the form of chemical compounds, also called corrosion products [1]. To prevent corrosion, many alloys have been developed; however, the use of these materials may not be practical from an economic point of view, depending on the specific application. However, less expensive materials, which have acceptable physical and mechanical properties, can be used if they are protected with anticorrosion coatings, even if metallic materials do not have the desired corrosion resistance [2].

Recently, so-called smart coatings have been developed for the corrosion protection of engineering alloys, which consists of adding corrosion inhibitors to a typical barrier coating [3]. This type of coating has two main objectives: (1) to increase corrosion resistance and to restrict its spread when it begins and (2) to avoid chemical interaction between the inhibitors and the components of the coating matrix formulations, as this may reduce the barrier properties of the final coating or lead to a complete deactivation of its inhibitory activity [4,5]. These concepts could be used in the protection of metallic implants or bioresorbable metallic components, constituting a recent field of investigation [6].

To accomplish both objectives, intelligent protection systems must provide corrosion inhibition when the physical barrier of the coating matrix is damaged, for example, via interaction with the environment or because of solar UV radiation, promoting the corrosion process [7]. In this regard, a practical and technological option is to encapsulate corrosion inhibitors in nanocontainers that respond to various stimuli, such as the presence of moisture, pH changes, chloride ion concentration, mechanical damage, or temperature changes. The result of this response is the release of the corrosion inhibitors in a controlled way, creating what is known as controlled-release systems (CRSs) [8,9]

CRSs with sensitivity to pH changes have been widely used in biomedical areas for controlled drug delivery [10]. They have also been used in the field of anticorrosive coatings since metallic corrosion is accompanied by localized pH changes [11]. Generally, at cathodic sites, there is an increase in pH, producing local alkalinization, while at anodic sites, there is a decrease in pH, leading to acidification [12]. Corrosion inhibitor molecules can modify the anodic reaction, the cathodic reaction, or both. One type of inhibitor recently used is the caffeine molecule, which has been reported to act as a cathodic inhibitor [13,14].

Recently, MCM-41 mesoporous silica nanoparticles (MSNs) have been used as nanocontainers, as they possess advantages, such as high surface areas and pore volumes, thermal and chemical stability, and biocompatibility [15,16,17,18,19]. However, by themselves, MSNs cannot constitute a CRS since they do not present any type of sensitivity; however, the great functionalization diversity possibilities of their surface offer a unique advantage in the construction of nanogates that respond to changes in the pH, creating four main types of functionalization with this sensitivity, namely, polyelectrolytes, supramolecular nanovalves, sensitive bonds, and polymers that coat the MSNs [20,21]. In this regard, poly(β-amino esters) are a group of biocompatible polymers that have recently attracted enormous attention [22,23,24] because they possess a key feature for their application in the sensitization of CRSs to pH variations, as it has been found that, at neutral pH, they are solid and stable while dissolving in acidic media [24]; moreover, due to these characteristics, they have recently been used to bind to MSNs and to create CRSs for anticancer drugs [25,26].

This research involved the synthesis and electrochemical evaluation of nanocontainers of a corrosion inhibitor sensitive to pH changes by means of encapsulating caffeine molecules in the mesoporous structure of MCM-41 MSNs, with sensitivity to pH changes provided by coating them with a PbAE finalized with APTES for covalent binding to the surface of the MSNs. Additionally, the inhibition capacity and efficiency of the caffeine encapsulated in the synthesized nanocontainers were studied and compared with the directly added free caffeine in the system of carbon steel in an acidic NaCl solution.

## 2. Materials and Methods

### 2.1. Materials

The materials were used as they were received without further purification. Hexadecyltrimethylammonium bromide (CTAB, Sigma ≥ 99%), tetraethyl orthosilicate (TEOS, Sigma ≥ 99%), sodium hydroxide (NaOH), 3-aminopropyl triethoxysilane (APTES, Sigma ≥ 99%), ethyl alcohol, hydrochloric acid, caffeine, 1-4 butanediol diacrylate, 4-4 trimethylendipiperidine, tetrahydrofuran (THF), and hexane were obtained from Sigma-Aldrich.

### 2.2. MSNs Synthesis

The MSNs were obtained using the sol–gel method. For this, 0.2 g of hexadecyltrimethylammonium bromide was mixed with 96 mL of distilled water and 0.7 mL of 2 M NaOH in a balloon flask, which was kept under heating and magnetic stirring until reaching 80 °C. Once this temperature was reached, a mixture of 1.4 mL of TEOS dissolved in 3 mL of ethanol was slowly added via dripping while magnetic stirring and heating at 80 °C continued for 2 h. At the end of this time, the solution was filtered and dried on a stove for 2 h. Finally, the MSNs were introduced to a muffle at 550 °C for 5 h in order to remove the surfactant. These samples were called MSNs.

### 2.3. Caffeine Encapsulation

For caffeine encapsulation, a previously reported method was followed [14]. Briefly, 50 mg of the MSNs was introduced into a vial-type tube and suspended in 5 mL of a 40,000 ppm caffeine solution; next, the vials were placed in an ultrasonic bath for 30 min and then left under mechanical agitation for 24 h. At the end of this time, the samples were centrifuged at 9000 rpm for 15 min, the supernatant was removed, and the products were placed on a stove for 2 h at 50 °C until complete drying. These materials were called MSNs-Caf.

### 2.4. PbAE Synthesis

Employing a Michael-type reaction, a PbAE was synthesized, combining 1-4 butanediol diacrylate and 4-4 trimethylenpiperidine in a stoichiometric ratio of 1.2:1 of diacrylate:amino; then, 5 mL of tetrahydrofuran (THF) was added to the mixture and left under magnetic stirring for 48 h at reflux with a temperature of 50 °C [24]. The prepolymer was precipitated via dripping in 60 mL of hexane under vigorous agitation; then, the solvents were removed via settling, and the polymer was dried at 60 °C on a stove, obtaining a prepolymer with terminal acrylate groups. This material was called PbAE.

The prepolymer of the PbAE obtained was finalized by suspending 1 g of this in 10 mL of THF and in 0.340 μL of 3-aminopropyltrietoxysilane (APTES) at 50 °C (24 h, 300 rpm) to add hydrolyzable siloxane groups and, subsequently, to bind them covalently to the MSNs. At the end of this time, the polymer with APTES was dripped in hexane, and the solvent was removed via settling in the same way as the prepolymer of PbAE, allowing it to dry at room temperature to obtain the polymer named PbAE-APTES.

### 2.5. Modification of MSNs with PbAE

**Scheme 1:**
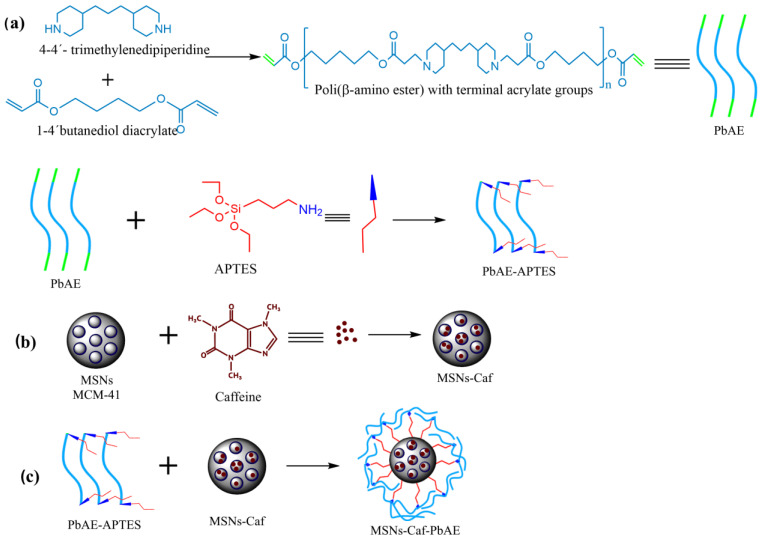
(**a**) Synthesis of the prepolymer and its modification with APTES, (**b**) encapsulation of caffeine in MSNs, and (**c**) modification of the MSNs-Caf with PbAE.

For the covalent binding of the PbAE-APTES to the MSNs-Caf, the surface silanol groups of the latter were reacted with the silanol groups of the APTES from the PbAE-APTES to obtain the nanocontainers for the controlled release of caffeine (Scheme 1). For this process, 50 mL of toluene and 1.2 g of PbAE-APTES were poured into a balloon flask, which was kept under magnetic stirring and reflux until the polymer was dissolved; finally, 1 g of MSNs-Caf was added, and the content of the balloon flask was left under agitation for 24 h. At the end of this time, the nanoparticles were precipitated, and the solvent was removed via settling. After this step, nanocontainers with pH sensitivity were obtained and called MSNs-Caf-PbAE.

### 2.6. Characterization of Nanocontainers

Scanning electron microscopy (SEM) images were obtained using a JSM-760 (JEOL) scanning electron microscope with an acceleration voltage of 10 kV. The particle diameter size was determined by taking the average value after measuring 100 MSNs. The functional groups of the MSNs and PbAE were analyzed in each of the stages of synthesis using attenuated total reflectance–Fourier transform infrared spectroscopy (ATR-FTIR) in a spectral range between 4000 and 650 cm^−1^, averaging 32 scans with a resolution of 4 cm^−1^, utilizing an FT-IR spectrometer model Agilent Cary 630. Small-angle X-ray diffraction (SAXRD) was used to determine the ordering of the mesopores from the MSNs and was performed using a Brunker D-8 Advance X-ray diffractometer with a CuKα of 1.542 Ǻ with a step of 0.01° at a step time of 0.5 s carried out in a low-angle interval of 2θ from 0.8° to 10°. A thermogravimetric analysis (TGA) was performed using Thermal Discovery Series TA Instruments equipment in a temperature range between 30 and 800 °C at a heating rate of 10 °C/min under a nitrogen atmosphere. 

### 2.7. Evaluation of Caffeine Release from MSNs Using UV–Vis Spectroscopy

The evaluation of the caffeine release from the MSNs was carried out using UV–Vis spectroscopy with a unique brand spectrophotometer model UV-2100. The analysis consisted of suspending the MSNs in 3.5% NaCl solutions (because this was the electrolyte used in the EIS analysis) with pH values adjusted to 4, 5, and 7, maintained under constant agitation. Next, 3 mL of the solution was taken at 1, 2, 3, 4, 5, and 24 h and then centrifuged at 9500 RPM for 10 min, and the supernatant was analyzed in the UV–Vis spectrometer at a wavelength of 273 nm [27]. At the end of the UV–Vis evaluation, each sample was returned to the release medium in order to maintain a constant volume until the end of the analysis.

### 2.8. Electrochemical Characterization

Electrochemical impedance spectroscopy (EIS) and potentiodynamic polarization were used to evaluate the behavior of the caffeine as a corrosion inhibitor and the electrochemical performance of the MSNs-Caf-PbAE nanocontainers. A conventional three-electrode electrochemical cell was used with a saturated calomel electrode (SCE) as the reference electrode, a graphite bar as the auxiliary electrode, samples of API X60 carbon steel as working electrodes, and a 3.5% NaCl solution with pH 5 at 26 °C as the electrolyte. The evaluation of the electrochemical behavior was carried out by adding the following to the NaCl solution: 0, 150, and 324 ppm of free caffeine and 324 ppm of caffeine encapsulated in the MSNs-Caf-PbAE. The latter concentrations were used because they are the concentrations of encapsulated caffeine present in a hypothetical coating when adding 0.2% *w*/*v* of MSNs-Caf-PbAE, which is a typical concentration for intelligent coatings [14]. It is of paramount importance to make a comparison of the behavior of free caffeine and that which is encapsulated within the nanocontainers. The working electrodes of the X60 carbon steel had an exposed area of 1 cm^2^, and the tests were carried out three times to ensure reproducibility. For EIS, the measurements were made in a frequency range of 20,000–0.002 Hz, applying a sinusoidal potential signal of 15 mV of amplitude. The potentiodynamic polarization was conducted by application of a cathodic overpotential of 400 mV vs. SCE and an anodic overpotential of 500 mV vs. SCE at a scan rate of 20 mV/min. The measurements were carried out on a Solartron Analytical 1280 °C electrochemical workstation, using a PC to control test programming and running.

## 3. Results and Discussion

### 3.1. Characterization of MSNs

**Figure 1:**
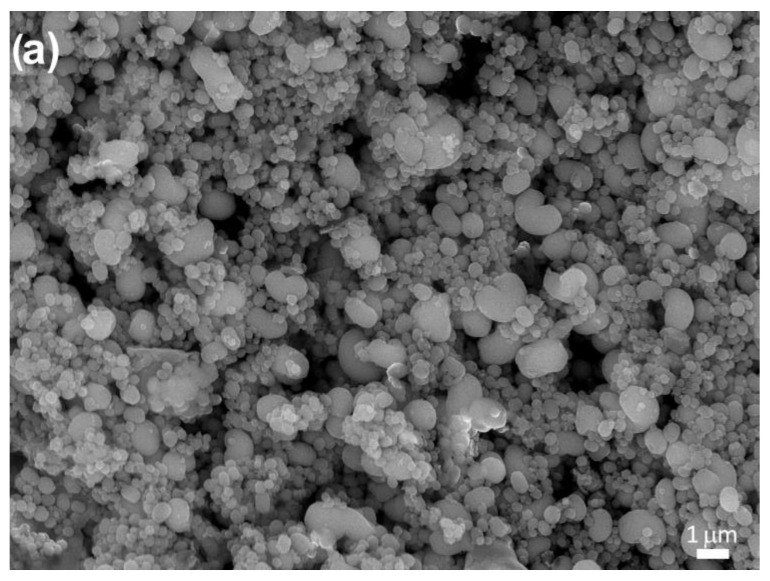

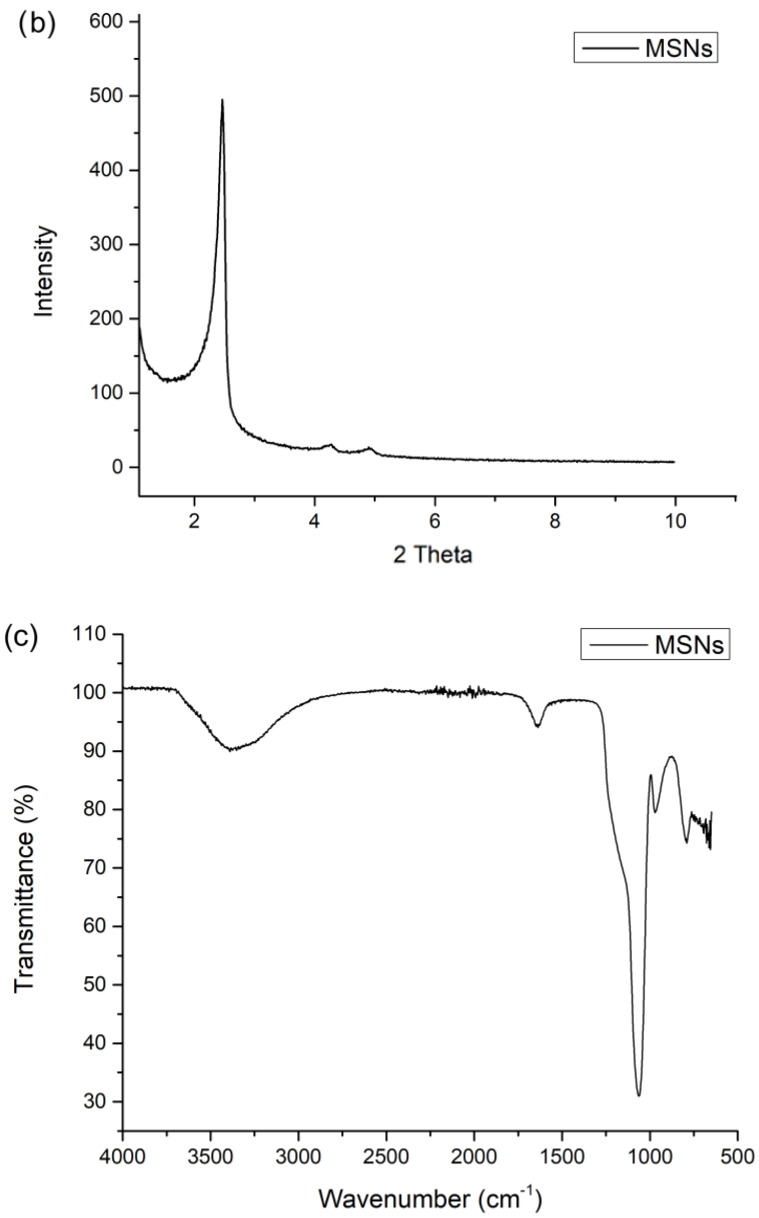
Characterization of the MSNs: (**a**) SEM image, (**b**) SAXRD diffractogram, and (**c**) FTIR spectrum.

The morphology of the MSNs was examined by performing an SEM analysis. As shown in Figure 1a, these materials present a nearly spherical morphology, data that coincide with those previously reported [28]. The diameter of the MSNs was measured to be an average of 301 ± 33 nm, which can be attributed to the concentration of NaOH or to the influence of the ethanol used in the synthesis, as was described in previous investigations [29,30,31].

As observed in the SAXRD diffractogram (Figure 1b), the MSNs show peaks corresponding to the (100), (110), and (200) atomic planes of the hexagonal mesoporous structure, indicating a high degree of ordering as previously reported in the literature [16]. It is possible to corroborate the presence of the MSNs via the FTIR spectra (Figure 1c), which show a peak associated with Si-O-Si asymmetric stretching at 1062 cm^−1^, as well as a signal located at 796 cm^−1^, which is attributed to the symmetrical vibration of the Si-O-Si bond of the silica network. The stretching of the OH groups is assigned to the signal at 3402 cm^−1^. A peak at 1632 cm^−1^ attributable to the bends of OH bonds can also be observed [32]. Additionally, there are no peaks located at 2930 cm^−1^ and 2850 cm^−1^, corresponding to the symmetrical and asymmetric tension-type vibrations of methylene groups, respectively, that could indicate the presence of surfactant, so its complete removal is confirmed [21,33]. 

### 3.2. Characterization of Nanocontainers

#### 3.2.1. FTIR

**Figure 2:**
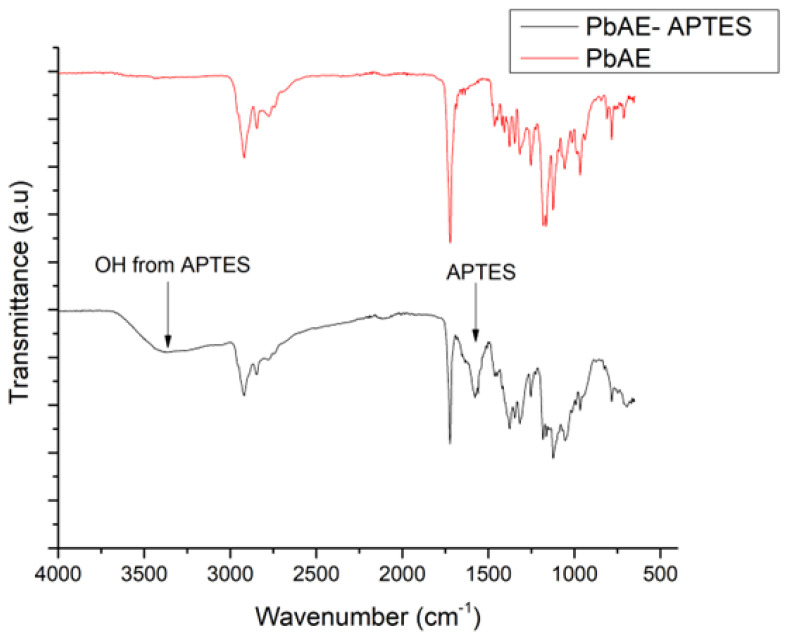
FTIR spectra of poly(β-amino esters) with and without APTES.

The first step for the synthesis of nanocontainers is the synthesis of PbAE and its APTES finalization (PbAE-APTES). The FTIR spectra obtained for these two samples are presented in Figure 2. The prepolymer shows the characteristic peaks of PbAE at 2930 and 2850 cm^−1^, which correspond to the asymmetric and symmetrical stretches of methylene groups, respectively; the peaks at ~2780 cm^−1^ are assigned to the stretching of the N-CH_2_ bond, confirming the presence of the tertiary amine groups of the 4,4- trimethylendipiperidine attached to the acrylate groups. The band near 1737 cm^−1^ is characteristic of a carbonyl ester group (C=O). Due to the stoichiometric relationship used during the synthesis, residual acrylate groups are present; this is confirmed via the band near 1400 cm^−1^, which is characteristic of flexion in the plane of the bond = C-H, while the peaks between 850 and 775 cm^−1^ are assigned to the bending and torsion bands of N-H [26,32,34]. 

The PbAE-APTES sample has an FTIR profile similar to that of PbAE; however, there is a band at 1578 cm^−1^ that corresponds to the amino groups of the APTES and is confirmed by the band located at 1636 cm^−1^ assigned to the APTES. Finally, the peaks at 966 cm^−1^ are those corresponding to the Si-O bonds, and the broad peak at 3400–3200 cm^−1^ corresponds to the OH groups of the hydrolyzed APTES, confirming that the APTES successfully grafted to the prepolymer [32,35].

**Figure 3:**
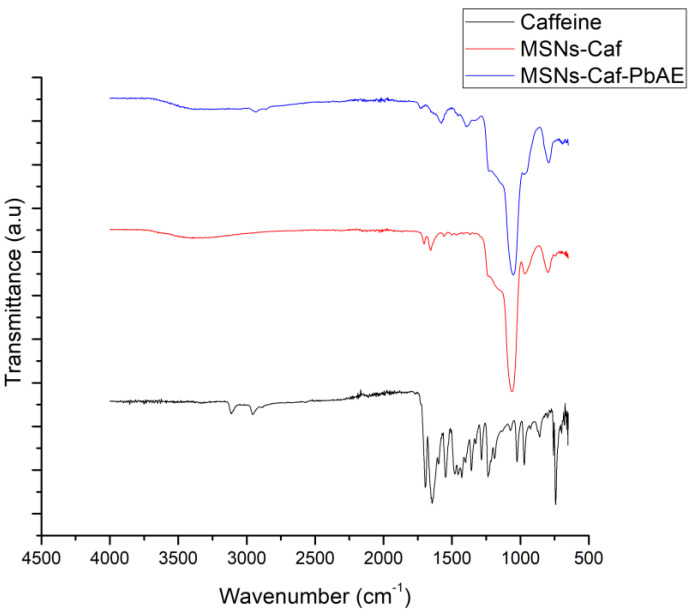
FTIR spectra of MSNs, MSNs-Caf, and MSNs-Caf-PbAE.

Figure 3 shows the spectra of the FTIR obtained from the caffeine, MSNs-Caf, and MSNs-Caf-PbAE. As can be observed, caffeine has a peak at 3112 cm^−1^ corresponding to the aliphatic C-H bond stretching vibration; at 2955 cm^−1^, there is a peak attributed to the -CH_3_ groups attached to nitrogen, while at 1639 and 1692 cm^−1^, characteristic peaks of carbonyl groups (C=O) are observed. Stretches of the double bond C=C also appear at 1548 cm^−1^ and 1478 cm^−1^; the peaks at 1283 and 1244 cm^−1^ correspond to the C-N bond, while the signal at 736 cm^−1^ corresponds to the C-H bond, and at 658 cm^−1^, a bending peak attributed to N-C=O is observed [27,32]. Likewise, caffeine can be identified in the MSNs-Caf sample by the two characteristic peaks located at 1654 cm^−1^ and 1705 cm^−1^ corresponding to carbonyl vibrations (C=O) (Figure 3). Additionally, in the MSNs-Caf-PbAE sample, the characteristic peak of the carbonyl group is presented at 1576 cm^−1^, confirming the presence of caffeine; however, the difference between the MSNs-Caf sample and the MSNs-Caf-PbAE sample is given by the peak shift from 1705 cm^−1^ to 1731 cm^−1^ because this wavenumber is characteristic of the ester group present in the PbAE, thus confirming its presence on the surface of the MSNs [26]. Another difference between these samples is that the MSNs-Caf does not show the characteristic bands of methylene (2950 and 2830 cm^−1^) while the MSNs-Caf-PbAE sample does due to the contribution of methylene groups that occur in large quantities in the polymer chain, confirming their contribution to these samples [33]. 

#### 3.2.2. TGA

**Figure 4:**
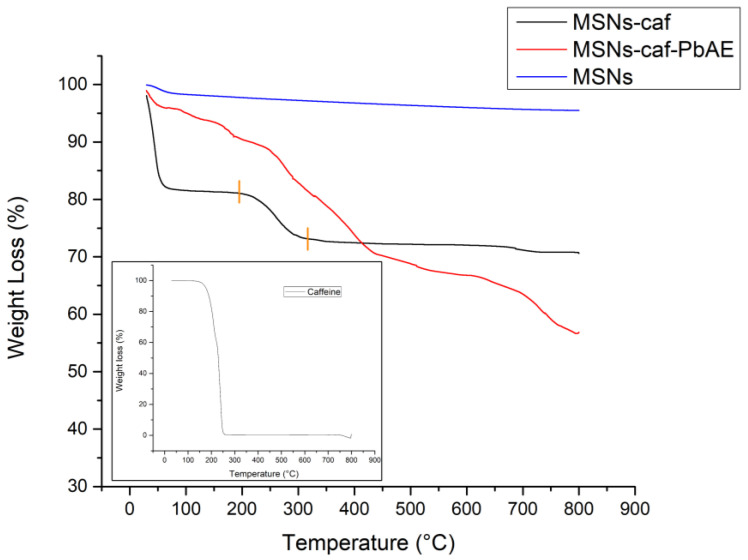
Thermogram curves of the caffeine sample (inset), MSNs, MSNs-Caf, and MSNs-Caf-PbAE.

A thermogravimetric analysis was carried out to evaluate the amount of caffeine encapsulation and to corroborate the modification of the MSNs with PbAE-APTES (Figure 4). As shown in Figure 4, the MSNs show a small weight loss of 4.4% corresponding to water removal and possible silanol decomposition. However, for the MSNs-Caf sample, the first weight loss was 16.6% at approximately 125.12 °C, consistent with the loss of water and low-molecular-weight molecules (e.g., ethoxy residual groups), as well as the condensation of silanols and possible presence of caffeine molecules on the surface of the MSNs. The second weight loss was 8.8% and occurred at 255 °C, which suggests that this is the amount of caffeine encapsulated in the MSNs, as it was confirmed by this same analysis that caffeine loses all of its mass at this temperature (inset Figure 4) [36]. These results suggest that the inhibitor encapsulation capacity in this research work is similar to the carrying capacity reported by other authors who found encapsulation values less than 10% [37,38,39]. Finally, the MSNs-Caf-PbAE sample had the greatest weight loss, losing a total of 42.1%, which is attributed to the great organic contribution of the PbAE, suggesting the correct addition of the polymer to the surface of the MSNs and supporting the data obtained via the FTIR [33,40].

### 3.3. Controlled Release of Caffeine in Solutions with Different pH Values

**Figure 5:**
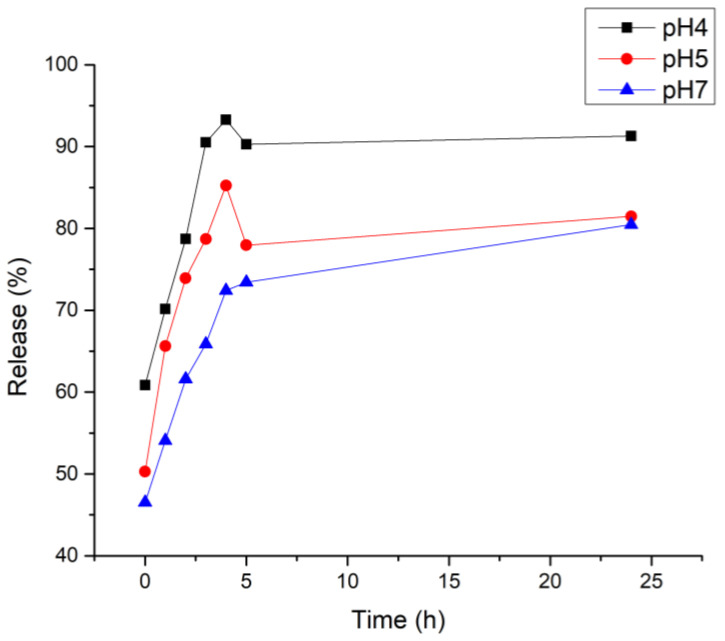
Comparison of the caffeine release profiles from MSNs-Caf-PbAE at different pH values.

Figure 5 shows the release profiles of the MSNs-Caf-PbAE at pH values 4, 5, and 7 at 24 h of analysis. All the samples showed their maximum percentage of release at 4 h and a reduction of this at 24 h, which coincides with previous reports [11]. This might be due to a possible re-encapsulation process due to the breaking of the polymer that covers the MSNs leaving the pores of these free again to store caffeine molecules. The releases at 4 h were 72%, 85%, and 93%, while those at 24 h were 80%, 81%, and 91% for the solutions with pH values of 7, 5, and 4, respectively. These results suggest that the MSNs-Caf-PbAE nanocontainers are sensitive to acidic media, releasing the encapsulated caffeine at 4 h and starting sensing at pH 5, and that the release of the inhibitor at acidic pH values occurs due to the action of the polymer on the surface of the nanocontainers because, at these pH values, the polymer expands as a result of the protonation of its amines, which allows the opening of the nanoparticle mesopores and, thus, the release of the corrosion inhibitor [24,26]. Therefore, it was confirmed that, at pH 5, the sensitivity effect of the nanocontainers begins.

### 3.4. Electrochemical Evaluation of Caffeine and MSNs-Caf-PbAE Nanocontainers as Corrosion Inhibitors Using Potentiodynamic Polarization and Electrochemical Impedance Spectroscopy

**Figure 6:**
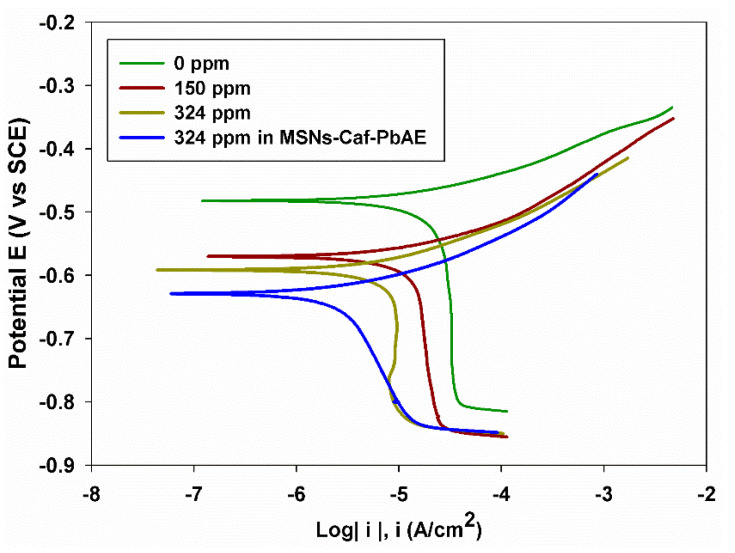
Polarization curves from the potentiodynamic polarization of the API X60 carbon steel in 3.5% NaCl solution with pH = 5, with direct addition of 0, 150, and 324 ppm of free caffeine compared with a solution containing 324 ppm of caffeine encapsulated into MSNs-Caf-PbAE (0.2% MSNs *w*/*v*) at 27 °C.

The experimental results of the potentiodynamic polarization tests in the form of polarization curves show that caffeine acts as a cathodic inhibitor because it effectively retarded the corrosion process that occurred in the carbon steel immersed in the 3.5% NaCl solution at pH = 5 at 27 °C, as shown in Figure 6 [13,41].

Figure 6 clearly shows that the corrosion potential of the carbon steel immersed in the NaCl solution changed to more negative values with the addition of caffeine. Despite the anodic and cathodic reactions presenting a similar tendency when compared with polarization without caffeine, the values of the current density decreased for both reactions. The anodic and cathodic current densities change to smaller values with the addition of higher concentrations of caffeine. As reported by A. A. Al-Amery et al., an inhibitor can be classified as an anodic or cathodic type when the change in the E_corr_ is > 85 mV [42,43]. It is worth mentioning that, for the potentiodynamic polarization using 324 ppm of caffeine encapsulated in the MSNs-Caf-PbAE (0.2% MSNs *w*/*v*), E_corr_ shifted to more negative values than in the free condition, and the anodic and cathodic current densities presented lower values. There is one order of magnitude difference between the cathodic current density of the carbon steel solution without the inhibitor and the system with the caffeine encapsulated. This shows the great potential of this controlled-release system for controlling corrosion in acidic media

**Figure 7:**
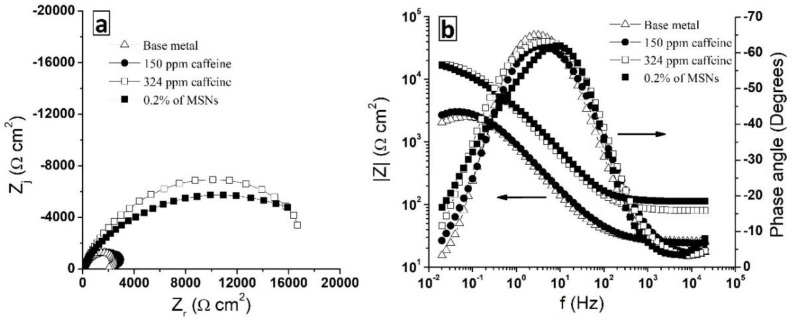
(**a**) Nyquist and (**b**) Bode plots (|Z|) and phase angle as a function of frequency for X60 steel immersed in pH 5 solution, 3.5% NaCl at 26 °C, with 0 ppm, 150 ppm, and 324 ppm caffeine and with 0.2% MSNs *w*/*v* (the arrows indicate the corresponding axe for each curve).

**Figure 8:**
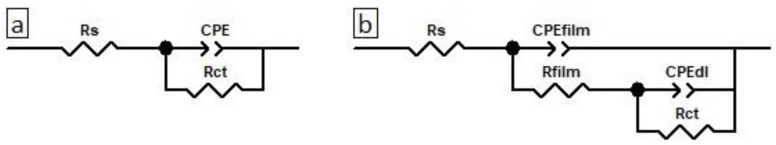
The equivalent electrical circuit used for fitting the experimental impedance data (**a**) in the presence and absence of inhibitor and (**b**) for the base metal/nanoparticle–caffeine system.

The EIS technique allowed us to determine the protection of free caffeine and caffeine encapsulated in the MSNs-Caf-PbAE as a corrosion inhibitor on API X60 steel. Figure 7 shows the electrochemical impedance spectra of the API X60 steel immersed in a 3.5% NaCl solution with pH = 5. This pH value was chosen because this is the pH at which polymer sensitivity was activated on the surface of the MSNs-Caf-PbAE, as observed in the UV–Vis release analysis. In the Nyquist and Bode diagrams, the experimental values are indicated by dot symbols, and the fitted behavior is presented as continuous lines. The fitting was conducted using the respective equivalent electric circuits (EECs), as shown in Figure 8. The EIS measurements on the carbon steel samples in contact with the 3.5% NaCl solution with pH = 5 were conducted in the caffeine-free solution with 150 ppm or o324 ppm of caffeine directly added to the electrolyte; and finally, with 0.2% *w*/*v* of MSNs-Caf-PbAE added to the electrolyte, whose encapsulated caffeine content was 324 ppm.

Figure 8a presents the EEC used for the fitting and interpretation of the measurement data of the system carbon steel—3.5% NaCl solution at pH 5 with 0, 150, and 324 ppm of caffeine. In this EEC, *R_s_* is the solution resistance; CPE is the constant phase element, which involves the parameters *Q* and *n*, representing the capacitance formed by the electrochemical double layer; and *R_ct_* corresponds to the charge transfer resistance. Figure 8b represents the EEC used for the fitting and interpretation of the system base metal/MSNs-Caf-PbAE (0.2% *w*/*v*), where *R_s_* is the resistance of the solution and CPE_film_ and *R_film_* correspond to the constant phase element and resistance of the film, respectively, which could be a combination of all species involved in the nanocontainer’s action, i.e., caffeine, PbAE broken chains, and/or MSNs deposited on the metal surface. CPE_dl_ represents the constant phase element of the double layer, and *R_ct_* represents the resistance to charge transfer. The values of the effective capacitance of the electrochemical double layer *C_dl_* (μF/cm^2^) (Equation (1)) [44] and the fractal dimension (*D*) (Equation (2)) were calculated [45], and they are presented in Table 1.

(1) $$C_{dl}={\left[Q_{dl}\;{\left(\frac{1}{R_{s}}+\frac{1}{R_{ct}}\right)}^{\left(n-1\right)}\right]}^{\left(\frac{1}{n}\right)}$$

where:

*R_s_* = solution resistance.
*R_ct_* = charge transfer resistance.
*n* = exponent.

However, the fractal dimension is

(2) $$D=\frac{1}{n}+1$$

where:

*D* = fractal dimension.
*n* = exponent.

**Table 1:** CPE parameters (*Q* and *n*), resistance, effective capacitance, and fractal dimension of the API X60 steel in 3.5% *w*/*v* NaCl solution, at pH = 5.0, in the presence and absence of caffeine as a corrosion inhibitor.

Caffeine Concentration ppm	*R_s_* Ω	$Q_{dl}$ Ω^−1^ cm^−2^ s^n^	*n*	*R_ct_* Ω cm^2^	*C_dl_* μF cm^−2^	*D*
0	25.35	1.34 × 10^−4^	0.74	3135	18.13	2.35
150	24.63	2.40 × 10^−4^	0.77	3495	51.72	2.29
324	78.54	6.97 × 10^−5^	0.77	19,435	14.69	2.29

Table 2 presents the parameters obtained by fitting the experimental data of the impedance spectra using the EEC shown in Figure 8b. For MSNs-Caf-PbAE, the effective capacitance of the electrochemical double layer *C_dl_* (μF/cm^2^) was calculated using Equation (3) [46]. The fractal dimension (*D*) was also calculated.

(3) $$C_{film}=Q_{film}^{1/n}\;\;\;R_{film}^{\left(1-n\right)/n}$$

where:

*C_film_* = film effective capacitance.
*Q_film_* = film capacitance.
*n* = exponent.
*R_film_* = film resistance.

**Table 2:** CPE parameters (*Q* and *n*), resistance, effective capacitance, and fractal dimension of API X60 steel in 3.5% *w*/*v* NaCl solution, at pH = 5.0, in the presence of 0.2% by weight of MSNs-Caf-PbAE as corrosion inhibitor.

*Rs* Ω	$Q_{film}$ Ω^−1^ cm^−2^ s^n^	*n*	*R_film_* Ω cm^2^	*C_film_* μF cm^−2^	$Q_{dl}$ Ω^−1^ cm^−2^ s^n^	*n*	*R_ct_* Ω cm^2^	*C_dl_* μF cm^−2^	*D*
112	3.90 × 10^−5^	0.86	4248	29.10	8.82 × 10^−5^	0.58	16,927	3.09	2.35

The coverage factor of the inhibitor coating on the surface of the metal at different concentrations, which is directly related to the efficiency of the inhibitor, was determined using Equation (4) [47]:

(4) $$\theta =\frac{i_{\mathrm{corr}-}i_{\mathrm{corr}\left(\mathrm{inh}\right)}}{i_{\mathrm{corr}}}$$

where:

θ = degree of inhibitory coating on the surface of the metal.
i_corr_ = corrosion rate without inhibitor (mA/cm^2^).
i_corr(inh)_ = rate of corrosion with inhibitor (mA/cm^2^).

According to the literature [48,49,50], the Rp values can be obtained from impedance spectra by calculating the equivalent resistance for each EEC: *R_ct_* for API X60 steel in 3.5% *w*/*v* NaCl solution in the absence and presence of caffeine (150 and 324 ppm) and *R_film_* + *R_ct_* for API X60 steel in 3.5% *w*/*v* NaCl solution in the presence of 0.2% by weight of MSNs-Caf-PbAE. i_corr_ was calculated using Equation (5):

(5) $$i_{\mathrm{corr}=}\frac{B}{\mathrm{Rp}}$$

where:

B = a constant of proportionality, which is equal to 0.026 V.
Rp = resistance to polarization (Ω cm^2^).

The values of the inhibition efficiency percentage, %*IE*, were calculated using Equation (6). R_p_, i_corr_, Θ, and %*IE* are presented in Table 3.

(6) %IE=Θ×100

In the Nyquist diagrams (Figure 7a), all systems presented a semicircle associated with a charge transfer process. The carbon steel immersed in the electrolyte without the inhibitor had Z_real_ values lower than 3500 Ω·cm^2^. Similar behavior was presented in the case of the steel in the electrolyte with 150 ppm of caffeine, observing *R_ct_* values very similar to those obtained in the measurements in the solution without the inhibitor. The values of i_corr_ and efficiency (10%), shown in Table 3, indicate that, when 150 ppm of caffeine was added to the electrolyte, no corrosion inhibition was produced. However, when 324 ppm of caffeine was added to the NaCl solution, considering that this was the concentration of caffeine in the MSNs-Caf-PbAE calculated with the encapsulation percentage, the corrosion inhibition effect was observed. In this case, Z_real_ increased to values close to 20,000 Ω·cm^2^. The latter can be confirmed with the impedance module vs. frequency diagram. Under these conditions, the inhibition efficiency increased up to 83.87%; therefore, it can be considered that the increase in the concentration of the caffeine changed the corrosion kinetics on the electrode surface. According to De Motte et al. [46], the charge transfer resistance (*R_ct_*) is considered to be inversely proportional to the active surface area (Sa), whereas the double-layer capacity (*C_dl_*) is directly proportional to Sa. As the amount of caffeine molecules deposited on the steel surface increased, the active surface area decreased, leading to an increase in *R_ct_* and a decrease in *C_dl_*, which suggests a decrease in the corrosion rate, as can be observed by the decrease in the i_corr_ values in Table 3. The decrease in the capacitance values of the double layer and the increase in the *R_ct_* values suggest that the MSNs-Caf-PbAE system functions as a barrier, conferring corrosion protection to the substrate and again corroborating that the MSNs-Caf-PbAE nanocontainers work properly to control the release of the inhibitor (Table 1 and Table 2).

The electrochemical behavior obtained for the caffeine contained in the smart nanocontainers was similar to that obtained for the system with 324 ppm of free caffeine, being appreciable in the Nyquist and Bode diagrams, which corroborate the correct function of the MSNs-Caf-PbAE as nanocontainers for the controlled release of the inhibitor caffeine. It should be noted that the values of icorr and efficiency for the MSNs-Caf-PbAE system were 1.22 μA cm^−2^ and 85.19%, respectively. The inhibition efficiency of the MSNs-Caf-PbAE system was slightly higher than that obtained in the solution with pure caffeine at 324 ppm.

Risović et al. [51] state that the fractal dimension lies within the range 2 ≤ D ≤ 3. A smooth surface has a value of D = 2, and an increasing value of D represents an increase in surface roughness/porosity. The value of the fractal dimension of 2.35 obtained for the steel in the absence of caffeine could be associated with the presence of heterogeneities on the metal surface caused by the corrosion of steel in the 3.5% *w*/*v* NaCl solution. The presence of caffeine in a solution, whose inhibition mechanism, according to Solehudin et al., involves adsorption on the metal surface [52], decreases the corrosion process of steel, which is reflected in a decrease in the fractal dimension value. We propose that the increase in the fractal dimension value for the MSNs-Caf-PbAE system was due to a combined effect between the nanocontainers, which have amine groups, making them attracted to the metallic surface, and either the caffeine molecules that act as an inhibitor depositing on the metallic surface or the possible deposition on the metal surface of broken polymer chain segments. This would increase surface inhomogeneities and, as a consequence, the value of the fractal dimension.

## 4. Conclusions

MCM-41 mesoporous silica nanoparticles were obtained via the sol–gel process, which was corroborated using SEM, FTIR, and SAXRD. In addition, using FTIR, the synthesis of PbAE and its finalization with APTES were corroborated. Additionally, the encapsulation of caffeine and the correct adhesion of the PbAE-APTES to the MSNs-Caf surface were corroborated with both (1) the appearance of specific peaks of caffeine and PbAE in the FTIR spectra and (2) the weight loss specific for caffeine in the MSNs-Caf sample and the maximum weight loss of the sample coated with PbAE due to the organic contribution of the polymer determined via a TGA analysis; all these results confirm the successful synthesis of the MSNs-Caf-PbAE nanocontainers for the controlled release of caffeine as a corrosion inhibitor.

These nanocontainers were demonstrated to preferably release the inhibitor molecules in an acidic medium with rapid action, releasing their maximum at 4 h, and their sensitivity was found to begin at pH 5 due to the action of the PbAE deposited on the surface of the MSNs-Caf-PbAE, which gave them the characteristic of sensitivity to acidic pH.

The analysis of the electrochemical behavior of the system under study using potentiodynamic polarization and electrochemical impedance spectroscopy measurements allowed us to conclude that free caffeine added to the electrolyte does work as a cathodic corrosion inhibitor for API X60 steel at concentrations of 150 and 324 ppm. Increasing the concentration of free caffeine to 324 ppm increased the inhibitor effect of the caffeine. The 324 ppm of caffeine corresponded with the quantity that was encapsulated in the MSNs-Caf-PbAE. The encapsulated caffeine in the MSNs-Caf-PbAE presented a higher inhibition efficiency (IE) than the same quantity of free caffeine added directly to the electrolyte which was 85.19%, demonstrating that these nanocontainers work correctly, releasing the corrosion inhibitor in the acidic NaCl solution and providing the medium with an inhibitory capacity. All the results obtained demonstrate the feasibility of using the synthesized MSNs-Caf-PbAE nanocontainers for the controlled release of caffeine as a corrosion inhibitor for the design and manufacture of smart coating applications.

## Figures and Tables

**Table 3:** Corrosion rate (i_corr_) and inhibition efficiency (%*IE*) of API X60 steel in 3.5% *w*/*v* NaCl solution, at pH = 5.0, in the presence and absence of caffeine as corrosion inhibitor and MSNs-Caf-PbAE.

Caffeine Concentration ppm	*R_p_* Ω cm^2^	i_corr_ μA cm^−2^	ϴ	%*IE*
0	3135	8.293	-	-
150	3495	7.439	0.103	10.30
324	19,435	1.338	0.838	83.87
MSNs-Caf-PbAE	21,175	1.228	0.851	85.19

## Data Availability

Data are contained within the article.
